# Preparation of Ion Composite Photosensitive Resin and Its Application in 3D-Printing Highly Sensitive Pressure Sensor

**DOI:** 10.3390/s25051348

**Published:** 2025-02-22

**Authors:** Tong Guan, Huayang Li, Jinyun Liu, Wuxu Zhang, Siying Wang, Wentao Ye, Baoru Bian, Xiaohui Yi, Yuanzhao Wu, Yiwei Liu, Juan Du, Jie Shang, Run-Wei Li

**Affiliations:** 1 School of Materials Science and Engineering, Shanghai University, Shanghai 200072, China; guantong@nimte.ac.cn; 2 Yongjiang Laboratory, Ningbo 315201, China; huayang-li@ylab.ac.cn; 3 CAS Key Laboratory of Magnetic Materials and Devices, Ningbo Institute of Materials Technology and Engineering, Chinese Academy of Sciences, Ningbo 315201, China; liujinyun@nimte.ac.cn (J.L.); zhangwuxu@nimte.ac.cn (W.Z.); wangsiying@nimte.ac.cn (S.W.); yewentao@nimte.ac.cn (W.Y.); bianbr@nimte.ac.cn (B.B.); yixiaohui@nimte.ac.cn (X.Y.); wuyz@nimte.ac.cn (Y.W.); liuyw@nimte.ac.cn (Y.L.); 4 Zhejiang Province Key Laboratory of Magnetic Materials and Application Technology, Ningbo Institute of Materials Technology and Engineering, Chinese Academy of Sciences, Ningbo 315201, China; 5 College of Materials Science and Opto-Electronic Technology, University of Chinese Academy of Sciences, Beijing 100049, China

**Keywords:** flexible pressure sensors, digital light processing, chitooligosaccharide, ion composite photosensitive resin, 3D printing

## Abstract

Flexible pressure sensors play an extremely important role in the fields of intelligent medical treatment, humanoid robots, and so on. However, the low sensitivity and the small initial capacitance still limit its application and development. At present, the method of constructing the microstructure of the dielectric layer is commonly used to improve the sensitivity of the sensor, but there are some problems, such as the complex process and inaccurate control of the microstructure. In this work, an ion composite photosensitive resin based on polyurethane acrylate and ionic liquids (ILs) was prepared. The high compatibility of the photosensitive resin and ILs was achieved by adding a chitooligosaccharide (COS) chain extender. The microstructure of the dielectric layer was optimized by digital light processing (DLP) 3D-printing. Due to the introduction of ILs to construct an electric double layer (EDL), the flexible pressure sensor exhibits a high sensitivity of 32.62 kPa^−1^, which is 12.2 times higher than that without ILs. It also has a wide range of 100 kPa and a fast response time of 51 ms. It has a good pressure response under different pressures and can realize the demonstration application of human health.

## 1. Introduction

Sensor technology plays an important role in modern science and technology [1,2]. Flexible pressure sensors can realize pressure sensing and are widely used in electronic skin [3,4], wearable devices [5,6], health monitoring [7,8,9], motion detection [10,11], and other fields [12,13]. According to the sensing mechanism, pressure sensors can be divided into four common types: resistive sensors, capacitive sensors, piezoelectric sensors, and triboelectric sensors [14,15]. A capacitive sensor that converts pressure stimuli into a capacitive signal has attracted wide attention due to its simple structure, ease to manufacture, low power consumption, and fast response [16]. However, the traditional capacitive sensor has a low sensitivity and small initial capacitance, which greatly limits its practical application [17,18]. In the past, researchers have overcome these problems by constructing microstructures [19]. The structure of the dielectric layer has a great influence on the sensitivity of the sensor. Compared with the dielectric layer without a microstructure, the sensitivity of the sensor assembled by the dielectric layer with a microstructure is significantly improved. Bao et al. [17] prepared polydimethylsiloxane (PDMS) films with pyramid microstructures by the photolithography silicon template method. Compared with the films without microstructures, the sensitivity of PDMS films with pyramid microstructures was increased by nearly 30 times. Guo et al. [20] used the plant template method to replicate the bionic microstructure from the lotus leaf, and had a high sensitivity of approximately 1.2 kPa^−1^, which was about 3.4 times higher than that without the microstructure. Shi et al. [21] used the electrospinning method to prepare the micro-cone array, and the sensitivity of the sensor was as high as 19 kPa^−1^, which was about 633 times higher than that of the two-dimensional planar structure film. However, these methods have the problems of a complex process and being time-consuming, and the microstructure cannot be accurately controlled, which cannot ensure the quality control of large-scale production. At the same time, these methods cannot construct complex three-dimensional structures, such as lattice structures [22].

Three-dimensional (3D) printing is also a common method for constructing microstructures. It can produce complex three-dimensional structures with controllable shapes by layer-by-layer printing [23,24,25]. Wu et al. [26] used direct ink writing (DIW) to print a hydrogel with a double network. The sensitivity of the network structure hydrogel reached 0.45 kPa^−1^ in the range of 0–1 kPa, which was five times higher than that of the block structure hydrogel. However, the nozzle of the equipment is easy to be blocked, and it cannot satisfy the preparation of high-precision microstructure sensors [27,28]. Different from DIW, 3D printing based on digital light processing (DLP) projects digital ultraviolet light onto the surface of liquid photosensitive resin, causing the local photopolymerization of the photosensitive resin to form a complex three-dimensional structure layer by layer [29]. DLP 3D printing has a high precision and fast speed, and can manufacture complex three-dimensional structures in a template-free manner, which can meet the quality control of mass production [22,30,31]. However, the application of DLP 3D printing in the fabrication of flexible pressure sensors is still limited by the photosensitive resin [32,33,34].

At present, the pressure sensor based on DLP 3D printing achieves a high sensitivity by adding ionic liquids (ILs) to form an electric double layer (EDL) [35,36,37,38]. Ge et al. [39] prepared a highly conductive ionic gel, and the assembled sensor achieved a high sensitivity of 15.1 kPa^−1^ and other excellent properties. In recent years, the ionic-gel-based elastomer prepared by introducing ILs into the polymer matrix has excellent stretchability, and the ionic sensor has the advantages of a high capacitance and excellent sensitivity [40,41]. However, there may be a poor compatibility between ILs and polymers, and it is difficult for ILs to enter the polymer network, resulting in a poor uniformity of the prepared sensors. And the Young’s modulus of the ionic gel will be greatly reduced with the increase in IL content, thus limiting the application detection range of the ionic sensors. At present, capacitive sensors based on DLP 3D printing have not shown significant sensing performance [16].

Here, this work reports a method for preparing an ion composite photosensitive resin. The photosensitive resin achieves hydrogen bonding and electrostatic interaction between ILs and oligomers by introducing a chitooligosaccharide (COS) chain extender, which improves the compatibility between the photosensitive resin and ILs. Then, a dielectric layer of lattice structure was designed. The designed structure was obtained by the DLP 3D printing of the ion composite photosensitive resin, and the capacitive sensor based on EDL was obtained by assembly. The sensor has a high sensitivity of 32.62 kPa^−1^, which is 12.2 times higher than that of the photosensitive resin without ILs. At the same time, the sensor is used for pulse monitoring and throat vibration detection, which realizes the feasibility of its demonstration application.

## 2. Experimental Section

### 2.1. Materials

The photosensitive resin with the main component of polyurethane acrylate used in this experiment was prepared in the laboratory and named PUA. Chitooligosaccharide (COS) was purchased from Shanghai Macklin Biochemical Technology Co., Ltd., Shanghai, China. Polyethylene Glycol (600) Dimethacrylate (PEG(600)DMA) was purchased from Sartomer (Guangzhou) Chemicals Ltd., Guanzhou, China. 1-ethyl-3-methylimidazolium bis(trifluoromethylsulfonyl)imide ([EMIM][TFSI]), 1-ethyl-3-methylimidazolium trifluoromethanesulfonate ([EMIM][OTF]), 1-ethyl-3-methylImidazolium Acetate ([EMIM]Ac), and isopropanol were purchased from Shanghai Aladdin Biochemical Technology Co., Ltd., Shanghai, China. X29 resin was purchased from Zhongshan Huayu Yuanxing Electronic Technology Co., Ltd., Zhongshan, China. eSUN resin was purchased from Shenzhen Guanghua Weiye Co., Ltd., Shenzhen, China. E600 resin was purchased from Zhongshan Dajian Technology Co., Ltd., Zhongshan, China. F80 was purchased from Shenzhen Shenshuo Technology Co., Ltd., Shenzhen, China.

### 2.2. Preparation of Ion Composite Photosensitive Resin

The ion composite photosensitive resin was prepared according to the following process, taking the addition of 40 wt.% ILs as an example. Next, 4 g COS and 16 g [EMIM][TFSI] were mixed and stirred to obtain a mixed solution. Then, 4 g PEG(600)DMA and the above mixed solution were added to 40 g PUA photosensitive resin in turn. After mixing at room temperature for 10 min, the multi-stage mode of the planetary mixer (ZYMC-200 V, Suzhou Zhongyi Precision Technology Co., Ltd., Suzhou, China) was used to stir and defoam. First, the equipment was run at 600 rpm for 30 s. Then, the vacuum condition of −100 kPa (relative to atmospheric pressure) was maintained for 60 s at a speed of 1500 rpm. Finally, keep this state running at 1000 rpm for 60 s. Thus, an ion composite photosensitive resin with uniform mixing and no bubbles is obtained.

### 2.3. Preparation of Dielectric Layer by DLP 3D Printing

The 3D model file was designed by LuxCreo (Qingfeng Technology Co., Ltd., Beijing, China) and imported into the slicing software to cut it into a multi-layer structure with a thickness of 25 μm and imported into the DLP 3D printer (AUTOCERAL, Beijing Shiwei Technology Co., Ltd., Beijing, China). The prepared resin was poured into the resin tank, and the ion composite photosensitive resin was cured for 3 s per layer under the ultraviolet light irradiation of 5 mW/cm^2^ (the curing power and time of the first few layers were appropriately increased). The printing layer thickness was 25 μm, and the printing temperature was set at 40 °C. After the printing was completed, the sample was placed in a mixed solution of isopropanol and ILs for ultrasonic cleaning for 2 min. After the sample was taken out, it was placed in a constant temperature and humidity box (BPS-50CL, Shanghai Yiheng Scientific Instrument Co., Ltd., Shanghai, China), and heated at a temperature of 80 °C and a relative humidity of 90% for 10 h. After drying, the printed sample can be used. It is worth noting that DLP 3D-printed samples have anisotropy in mechanical properties [42]. In order to improve the efficiency of printing, we use the largest area of the printed sample as the bottom of the printing to contact the forming table, and the thickness direction of the sample is the z-axis direction of the printing.

### 2.4. Preparation of Pressure Sensor

The Cu electrode used in the sensor was purchased from Shenzhen Jialichuang Technology Group Co., Ltd., Shenzhen, China. The substrate is PI, and the effective electrode area is 1 cm × 1 cm. After the prepared dielectric layer is aligned with the upper and lower electrodes, the final assembly of the sensor is completed by medical tape.

### 2.5. Characterization and Measurements

The Instron universal testing machine (5943) was used to apply a tensile force or pressure to the sample. The electrical signal output by the sensor was tested using an impedance analyzer (IM 3570, HIOKI Impedance Analyzer, Shanghai, China). The contact angle measuring instrument (OCA25, Dataphysics Co., Ltd., Beijing, China) was used to test the affinity of the photosensitive resin and ILs. The distribution of ILs in the dielectric layer was characterized by high-resolution scanning electron microscopy (SEM7, HITACHI, Tokyo, Japan). The composition of the material was tested by micro-infrared spectrometer (Cary660+620, Agilent, Beijing, China). The dielectric layer was tested by dual-electric four-probe tester (RTS-9, Guangzhou Four-probe Technology Co., Ltd., Guangzhou, China). The thermal stability of the material was tested by thermogravimetric analyzer (TG209F1, Naichi, Selbu, Germany). The viscosity of the ion composite photosensitive resin was tested by Discovery Hybrid Rheomete (HR-2, TA Instruments, Newcastle, USA). The response of the capacitive pressure sensor is defined as ΔC/C_0_, where ΔC (i.e., C-C_0_) refers to the relative capacitance change difference between the capacitor C after applying pressure and the initial capacitor C_0_ under no load pressure. The sensitivity of the capacitive pressure sensor is defined as δ(ΔC/C_0_)/δP, where δP is the relative change in loading pressure in the linear response range [43].

## 3. Result and Discussion

### 3.1. Fabrication and Characterization

**Figure 1:**
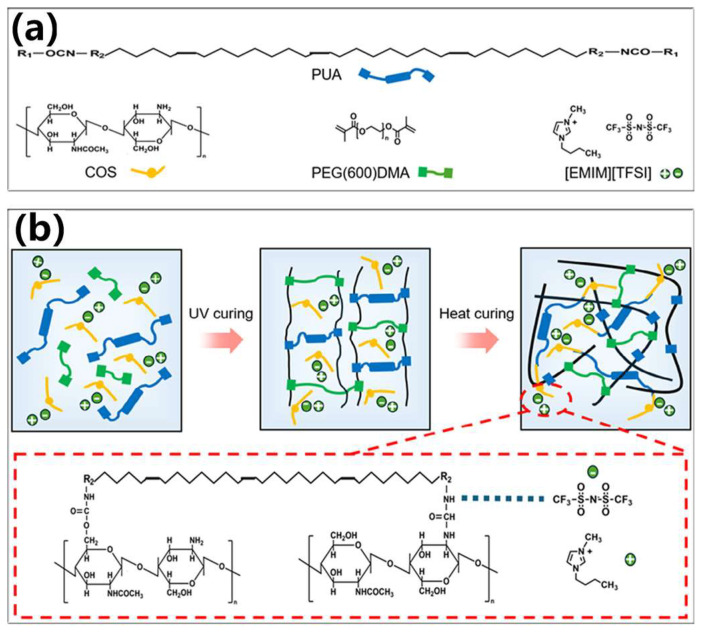
Curing principle of ion composite photosensitive resin: (**a**) chemical structure of PUA, COS, PEG(600)DMA, and [EMIM][TFSI]; and (**b**) the schematic diagram of UV curing and heat curing.

As shown in Figure 1a, the ion composite photosensitive resin that can be used for DLP 3D printing is composed of PUA photosensitive resin (including photoinitiator), COS, PEG(600)DMA and [EMIM][TFSI]. Figure 1b is the curing principle of the ion composite photosensitive resin. Firstly, under UV light irradiation, the photoinitiator in the PUA photosensitive resin absorbs light to produce free radicals, which initiates the polymerization of C=C to form an ultraviolet (UV) curing crosslinking network. Then, during the heat curing process, the protective group of -NCO of polyurethane acrylate in PUA is disconnected, thereby completing the chain extension of -NCO group by COS, forming a dual crosslinking network of UV curing and heat curing.

For polyurethane acrylate resins, some characteristic groups are often introduced by adding chain extenders to improve the properties of photosensitive resins [44]. COS has a good biocompatibility. There are a large number of active functional groups such as amino and hydroxyl groups in COS molecules, which can realize the chain extension of polyurethane acrylate [45]. In order to achieve the uniform distribution of ILs in the polymer network of photosensitive resin, COS is an effective and easy-to-obtain chain extender to improve the compatibility between the photosensitive resin and ILs. The principle is mainly due to the addition of COS to introduce a large number of -NH bonds, and, because COS has a positive charge, there are hydrogen bonds and electrostatic interactions between COS and ILs. Therefore, the high compatibility of the photosensitive resin and ILs and the uniform distribution of ILs are realized. Moreover, COS completed the chain extension reaction with PUA during the heat curing process. Figure S1 shows the FTIR spectra of two different materials after UV curing and the FTIR spectra of PUA after adding COS under different curing methods. It can be seen that, after the addition of COS, and only UV curing, the characteristic peak of the photosensitive resin changed little (Figure S1a). However, after heat curing, the characteristic peaks of -NH and C=O changed, indicating that COS reacts with -NCO in the process of heat curing. The specific reaction is shown in Figure S2. The PUA and COS completed the chain extension reaction during heat curing to generate -NH and C=O. The results of this reaction correspond to the FTIR spectra. Three commonly used ILs were selected for the contact angle test (Figure S3). After the addition of COS, the contact angles of the photosensitive resin and the three ILs decreased, indicating that the addition of a COS chain extender can indeed improve the affinity between ILs and the photosensitive resin. Among them, the contact angle between [EMIM][TFSI] and the photosensitive resin is the smallest, indicating that it has the best affinity with PUA, and this IL is selected for subsequent experiments.

**Figure 2:**
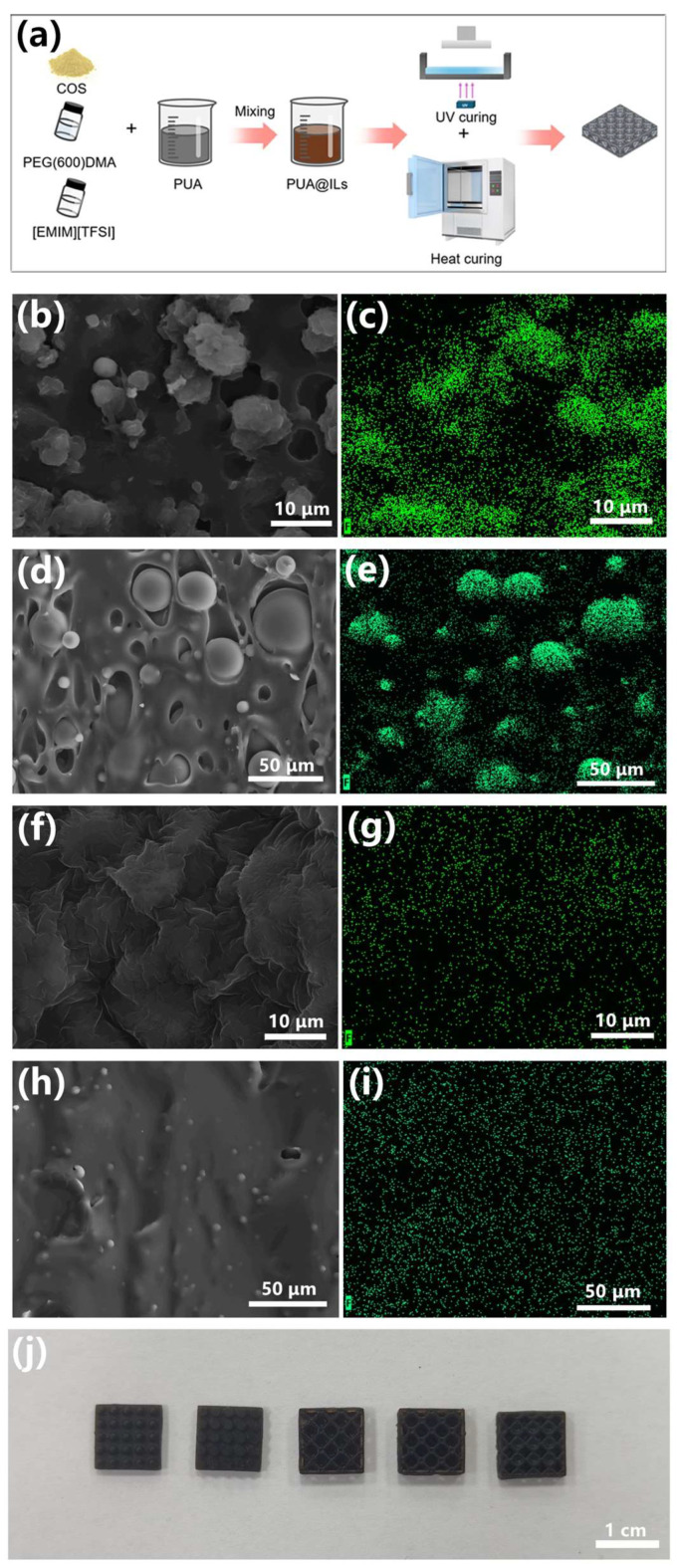
(**a**) Preparation process of dielectric layer; (**b**,**c**) SEM and EDX on the surface of ion composite photosensitive resin without COS; (**d**,**e**) the cross-section SEM and EDX of ion composite photosensitive resin without COS; (**f**,**g**) SEM and EDX on the surface of ion composite photosensitive resin with COS; (**h**,**i**) the cross section SEM and EDX of ion composite photosensitive resin with COS; and (**j**) image of different structures by DLP 3D printing.

The preparation method of the sensor dielectric layer is shown in Figure 2a, and the dielectric layer is prepared by UV curing and heat curing. We use this method to prepare 500 μm films of the photosensitive resin with different components, and the microscopic morphology of the surface and cross-section was observed. The F element is a unique element in ILs, and the distribution of the F element in the EDX image can help us judge the distribution of ILs. As shown in Figure 2b–e, it can be inferred that the balls in Figure 2b,d are ILs. When COS was not added, the ILs could not be evenly distributed in the sample. From the EDX, it can also be seen that the unique F element in [EMIM][TFSI] is unevenly distributed. After the addition of COS, the microstructure is shown in Figure 2f–i, and it can be seen that the distribution of ILs in the photosensitive resin is more uniform. The complex three-dimensional structure was 3D-printed by the ion composite photosensitive resin. The surface morphology of the structure and the distribution of ILs were analyzed by SEM and EDX. It can be seen from Figure S4 that ILs can still be uniformly distributed in the three-dimensional structure. This is mainly because, after the introduction of COS, there are more intermolecular interactions between the photosensitive resin and ILs, and the interaction between the photosensitive resin and ILs can be verified by FTIR spectra. As shown in Figure S5, compared with the photosensitive resin without ILs, the -NH stretching peak of the photosensitive resin after adding 40 wt.% ILs (subsequently named PUA@ILs) moved to a larger wavenumber, which is mainly because the hydrogen atoms in the polyurethane acrylate and COS form hydrogen bonds with the electronegative atoms in the ILs. At the same time, because the protonation of the N-H bond is positively charged, it can attract negatively charged anions through electrostatic interaction. Thereby improving the compatibility of photosensitive resin and ILs, ILs can be uniformly dispersed in the polymer network. The ion composite photosensitive resin with COS was used for DLP 3D printing. As shown in Figure 2j, this ion composite photosensitive resin can prepare a variety of complex three-dimensional structures.

### 3.2. Properties of Ion Composite Photosensitive Resin

**Figure 3:**
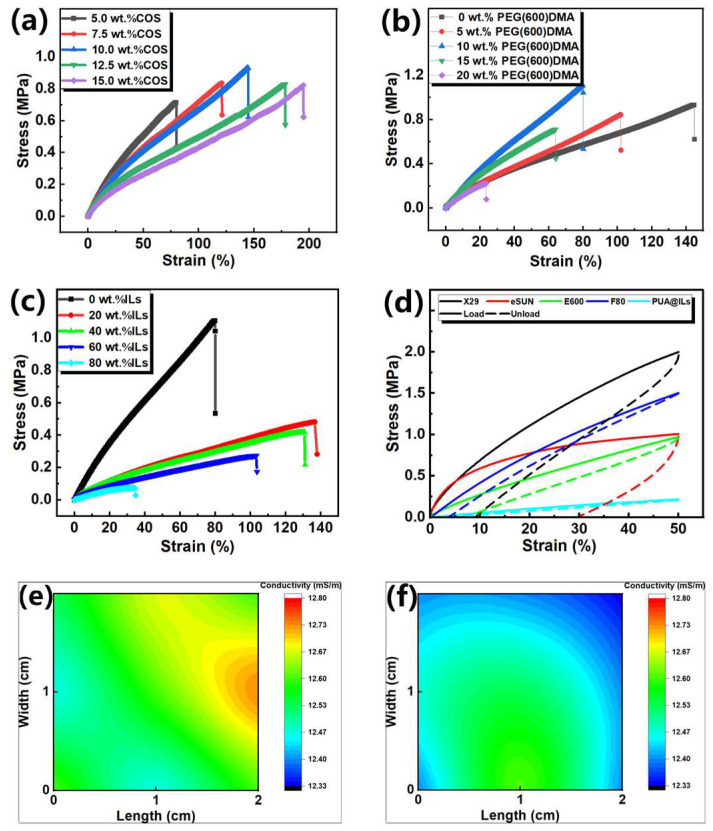
Mechanical and electrical properties of ion composite photosensitive resin: (**a**–**c**) the mechanical properties changed with the content of COS, PEG(600)DMA, and ILs; (**d**) the comparison of the tensile loading–unloading curves of the ion composite photosensitive resin containing 40 wt.% ILs (PUA@ILs) and other commercial flexible photosensitive resins; and (**e**,**f**) distribution of sample conductivity before and after compression.

After determining the preparation process of the material, the effects of COS, PEG(600)DMA, and [EMIM][TFSI] on the mechanical properties of the ion composite photosensitive resin were investigated. As shown in Figure S6a, the stress–strain response of the resin was tested using the tensile test mode of the Instron universal testing machine. The tensile sample was the type 3 sample specified in [46]. Its sample parameters are shown in Figure S6b. The thickness of the sample is 1 mm. We clamped the sample on the fixture of the testing machine, and the tensile displacement speed was set to 5 mm/min to test the sample. All samples in Figure 3a–d were tested by the same method. As shown in Figure 3a–c, the effect of COS chain extender, PEG(600)DMA crosslinking agent, and [EMIM][TFSI] on the mechanical properties of ion composite photosensitive resin was investigated. The first is to explore the effect of COS on the mechanical properties of the material. At this time, the material components include PUA and COS. It can be seen from Figure 3a that, when the content of COS increased from 5 wt.% to 15 wt.% (the weight percentage of the added material mentioned later is relative to the weight of PUA), the elongation at break of the material increased from 80% to 195%, and the Young’s modulus decreased from 1.46 MPa to 0.98 MPa. In order to balance the mechanical properties of the material, 10 wt.% COS was selected for subsequent experiments, which made the material have the highest tensile strength. Since the addition of ILs will greatly reduce the Young’s modulus of the photosensitive resin, it is necessary to increase the Young’s modulus of the material by adjusting the content of PEG(600)DMA (Figure 3b). By selecting the optimal composition ratio obtained in Figure 3a, the effect of PEG(600)DMA on the mechanical properties of the material was further explored. By regulating the content of PEG(600)DMA crosslinking agent from 0 wt.% to 20 wt.%, it is found that the increase in crosslinking agent content will lead to the decrease in elongation at the break of the material, and the Young’s modulus shows a trend of increasing first and then decreasing. It may be that, when a small amount of crosslinking agent is added, the molecular weight of the material becomes larger after curing, and the Young’s modulus becomes larger. After adding too much crosslinking agent, some small molecule crosslinking agents will not participate in the reaction and remain in the polymer network, reducing the mechanical properties of the material. When the content of PEG(600)DMA is 10 wt.%, the Young’s modulus reaches the maximum, so this ratio is selected to continue the follow-up experiment. At this time, the Young’s modulus of the photosensitive resin is 2.02 MPa, and the elongation at break is 80%.

**Figure 4:**
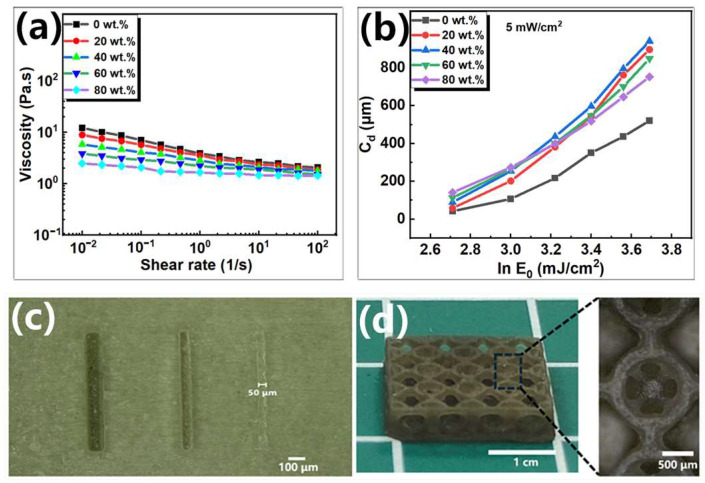
3D printing of ion composite photosensitive resin: (**a**) relationship between viscosity of photosensitive resin and content of [EMIM][TFSI]; (**b**) the relationship between curing depth and exposure energy; (**c**) horizontal resolution of ion composite photosensitive resin; and (**d**) image of TYPE-C structure printed by ion composite photosensitive resin.

[EMIM][TFSI] also has a great influence on the mechanical properties of the photosensitive resin. The optimal ratio of PUA, COS, and PEG(600)DMA was selected, and ILs were added to explore the effect of ILs on the mechanical properties of the material. As shown in Figure 3c, the increase in [EMIM][TFSI] content from 0 wt.% to 80 wt.% leads to a decrease in Young’s modulus from 2.02 MPa to 0.31 MPa. Four kinds of commercial resins sold on the market were selected. Comparing the ion composite photosensitive resin with 40 wt.% ILs with these commercial flexible photosensitive resins (Figure 4d), it can be seen that this ion composite photosensitive resin has a good recovery and small hysteresis curve area. At the same time, the content of [EMIM][TFSI] also has a certain influence on the conductivity of the ion composite photosensitive resin. As shown in Figure S7, the conductivity increases from 6.54 mS/m to 21.3 mS/m with the increase in content. In order to balance the conductivity and mechanical properties of the material, when the IL content is 40 wt.%, the cured ion composite photosensitive resin has a good Young’s modulus (0.85 MPa) and a suitable ionic conductivity (12.6 mS/m), so this ratio is used for subsequent experiments. The ion composite photosensitive resin was prepared by DLP 3D printing to obtain a cuboid of 20 × 20 × 1 mm. It was compressed and cycled for one hundred times under 30% strain by a fatigue machine to obtain a distribution map of conductivity before and after compression. We uniformly selected nine points on the film and tested their conductivity by a dual-electric four-probe tester. The current range during the test was set to 10 μA, the sample thickness was 1 mm, and the average probe spacing was 1 mm. We calculate the average value of each point after multiple measurements of conductivity and import it into Origin 2021. Through Origin 2021 processing, the conductivity data are gridded into 100 × 100 regular distribution data, and the conductivity mapping image of the film is obtained. As shown in Figure 3e,f, it can be seen that, before and after compression, the ILs were evenly distributed in the photosensitive resin, and the conductivity remained basically unchanged before and after compression (the average conductivity before compression was 12.6 mS/m, and it was 12.4 mS/m after compression), indicating that the photosensitive resin had a good stability. The mechanical properties of the samples before and after the fatigue test were tested. It can be seen from Figure S8 that the stress–strain curves of the samples basically coincide, indicating that the samples have a good mechanical stability. And the ion composite photosensitive resin showed excellent stability in a wide temperature range (Figure S9). When the temperature increased from room temperature to 250 °C, no obvious weight loss was observed, and the material had a good thermal stability.

For the photosensitive resin, in order to quickly level in the resin tank for the next layer of digital UV curing, the photosensitive resin should also have a lower viscosity. As shown in Figure 4a, the viscosity of the photosensitive resin was tested. It can be seen that the viscosity of the ion composite photosensitive resin decreased with the increase in IL content. The above photosensitive resin can be used for DLP 3D printing. The photosensitive resin was dropped on the glass slide, and the exposure power of 5 mW/cm^2^ was fixed by the DLP 3D printer. The exposure time range was 3 s to 8 s. The photosensitive resin was exposed to UV light. The uncured resin was washed by isopropanol, and the thickness of the cured resin was tested by a thickness gauge. Two key parameters of the photosensitive resin in the process of photopolymerization were calculated: E_c_ (the critical energy to initiate polymerization) and D_p_ (the penetration depth of the curing light). As shown in Figure 4b, the change in curing depth of the photosensitive resin with a different IL content with curing energy was tested respectively. It can be calculated that the E_c_ of photosensitive resin decreases with the increase in IL content. The possible reason is that ILs are colorless, and, when ILs are added, the light transmittance of the photosensitive resin becomes better, resulting in the better photosensitivity of the photosensitive resin. The D_p_ of the photosensitive resin increases first and then decreases with the increase in IL content. The possible reason is that, when a small amount of ILs is added, the transmittance of the photosensitive resin becomes better, resulting in a larger D_p_. However, as the content of ILs continues to increase, it may increase the scattering and absorption of UV light, resulting in a decrease in D_p_ (Figure S10). As shown in Figure 4c, the minimum horizontal resolution of the ion composite photosensitive resin can reach 50 μm. Using it to print complex three-dimensional structures, such as TYPE-C structures, it shows that microstructures can be accurately printed.

### 3.3. Sensing Properties

**Figure 5:**
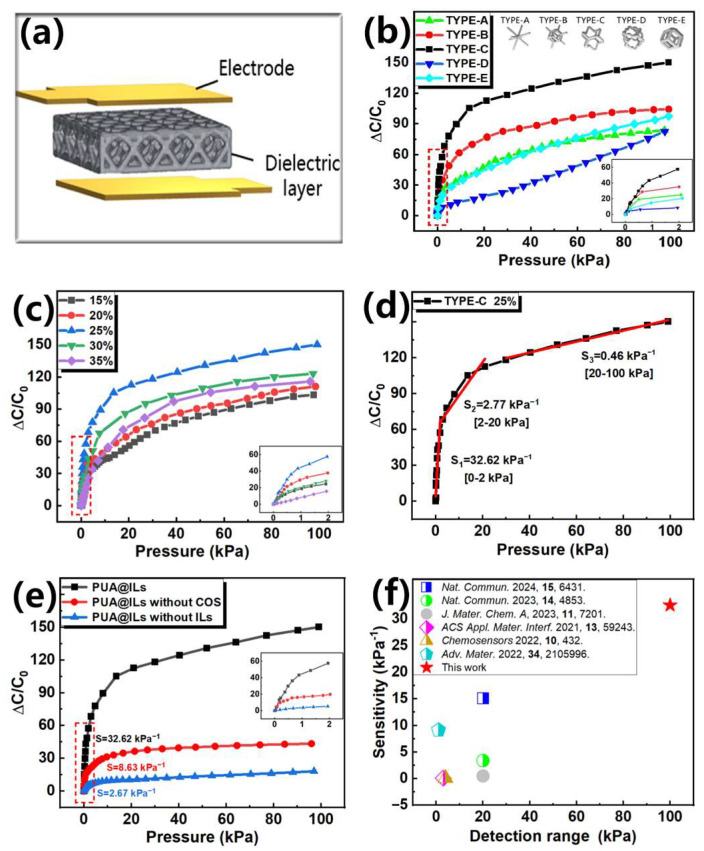
Sensing performance of the 3D-printed capacitive sensor based on ion composite photosensitive resin: (**a**) sensor structure diagram; (**b**) the ΔC/C_0_–P relation of sensors with dielectric layers of different lattice structures; (**c**) the ΔC/C_0_–P relation of sensors with TYPE-C dielectric layers of different density ratios; (**d**) the ΔC/C_0_–P relation of TYPE-C structure with 25% density ratio; (**e**) the sensitivity of sensors with different dielectric layer materials; and (**f**) comparison of sensitivity and range (sensitivity > 0.1 kPa^−1^) of different capacitive sensors based on photosensitive resin [32,37,38,39,47,48].

The dielectric layer is assembled with upper and lower electrodes to obtain a sensor. The structure is shown in Figure 5a, and the sensing performance of the sensor is tested. The principle of capacitive pressure sensor is to convert the change in pressure into the change in capacitance. Due to the addition of ILs, the ion composite photosensitive resin is prepared into a dielectric layer, and the sensor can form an EDL capacitance with high capacitance. This is because, when the voltage is applied, the positive and negative charges on the two electrodes will attract the negative and positive ions in the dielectric layer, respectively, forming many ion–electron pairs. At this time, the distance between electrons and ions is nanoscale, thus forming an iontronic sensor with an ultra-high capacitance. Different from the traditional capacitive sensor, the capacitance of the iontronic sensor increases significantly with the increase in the contact area between the electrode and the dielectric layer, rather than mainly depending on the change in the distance between the two electrodes [16].

The ion composite photosensitive resin can obtain various complex three-dimensional structures through DLP 3D printing. In order to manufacture a high-performance iontronic pressure sensor, it is necessary to improve the sensitivity of the sensor by adjusting the structure of the dielectric layer. At the same time, the structure obtained by DLP 3D printing also needs to have a high resilience to ensure that the detected signal has a good stability and repeatability. The common structure of the dielectric layer of the iontronic sensor is selected, as shown in Figure S11, in which the lattice structure has the best recovery and a small hysteresis area, so the lattice structure is selected to continue the follow-up experiment. Five common lattice structures in LuxCreo are selected. By controlling these lattice structures with the same linewidth and lattice size, five different lattice structures are obtained. The sensing performance of the sensors composed of these dielectric layers was tested. The TYPE-C structure has the best sensitivity, as shown in Figure 5b, which can reach 32.62 kPa*^−^*^1^ [0–2 kPa]. The enlarged view in the range of 0–2 kPa is shown in the illustration Therefore, this lattice structure is further optimized.

**Figure 6:**
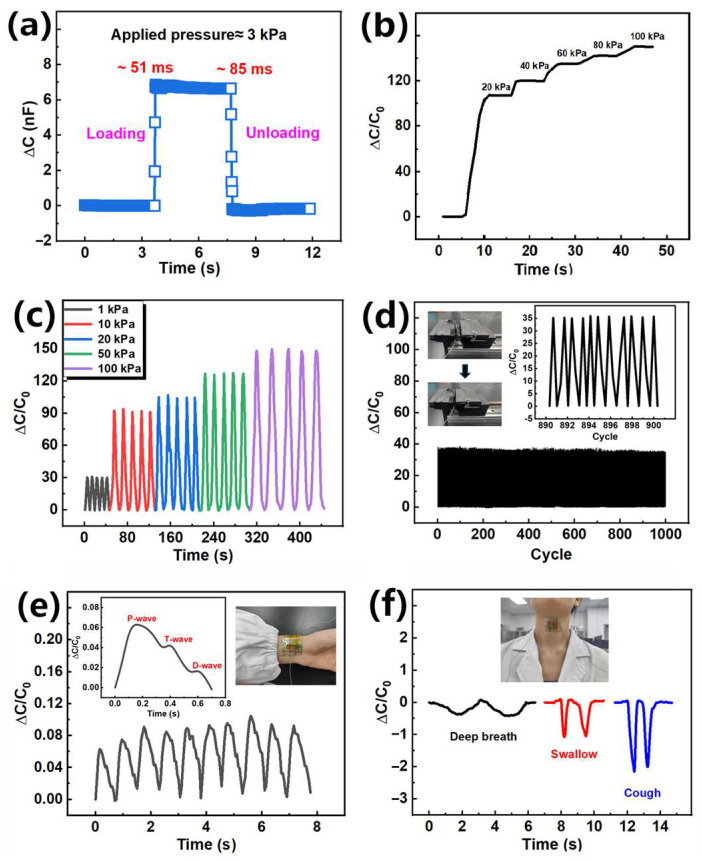
Other sensing performance and application demonstrations of the 3D-printed capacitive sensor based on ion composite photosensitive resin: (**a**) response time of the sensor at approximately 3 kPa; (**b**) response of the sensor to a gradually increasing force of 20 kPa; (**c**) the cyclic response of the sensor under different pressures (1 kPa, 10 kPa, 20 kPa, 50 kPa, and 100 kPa); (**d**) response of the sensor under 1000 cycles; (**e**) real-time monitoring of human pulse; and (**f**) real-time monitoring of deep breath, swallow, and cough in the human throat.

By controlling the line width of the TYPE-C structure, the density ratio of the dielectric layer structure is controlled to be 15%, 20%, 25%, 30%, and 35%, respectively, and the sensitivity of the sensor assembled by these dielectric layers is measured. As shown in Figure 5c and Figure S12, when the density ratio of TYPE-C structure is 25%, the sensitivity of the sensor is the highest, reaching 32.62 kPa*^−^*^1^ [0–2 kPa], and the detection range can reach 100 kPa (Figure 5d). When the linewidth of the dielectric layer is small, the change in the contact area between the dielectric layer and the electrode is small, resulting in a low sensitivity. Further increasing the linewidth will lead to a larger compression modulus of the dielectric layer. Under the same stress, the dielectric layer is difficult to compress, resulting in a decrease in sensitivity. The dielectric layer structure of the sensor is a lattice structure formed by the regular arrangement of small lattices. The TYPE-C structure with a density ratio of 25% is used as the dielectric layer for mechanism analysis. As shown in Figure 4d, the columnar skeleton of the lattice structure has a diameter of 240 μm. When the external pressure is applied, due to the good compressibility of the lattice structure, the electrode first contacts the columnar line at the top of the dielectric layer in the lower pressure range. With the action of pressure, the contact area gradually expands from the initial line to the surface, and the contact area increases significantly, resulting in a significant increase in capacitance. Therefore, the sensor exhibits a high sensitivity in the pressure range of 0–2 kPa. As the pressure further increases, the compressible range of the lattice structure gradually decreases, resulting in the need to apply more pressure to make the electrode contact other columnar skeletons of the dielectric layer. Therefore, when the pressure range is 2–100 kPa, the sensitivity of the sensor gradually decreases. The ion composite photosensitive resin containing 40 wt.% ILs (PUA@ILs), PUA@ILs without COS, and PUA@ILs without ILs were printed into the TYPE-C structure dielectric layer with a density ratio of 25% by DLP 3D printing. In the absence of external pressure, the impedance analyzer is used to read the capacitance value of the flexible pressure sensor assembled by these dielectric layers, and the capacitance at this time is the initial capacitance. When the ion composite photosensitive resin was not added with ILs as the dielectric layer, the initial capacitance of the sensor was only 6.48 pF. When ILs were added, the initial capacitance of the sensor increased significantly to 89.2 pF. It can be seen from Figure 5e that the ion composite photosensitive resin obtained in this work has the highest sensitivity, which indicates that COS and ILs can significantly improve the sensitivity of the sensor. This may be due to the addition of ILs, which caused the sensor to form an EDL capacitance, resulting in a significant increase in the capacitance of the sensor. At this time, the capacitance change of the sensor mainly depends on the change in the contact area between the electrode and the dielectric layer rather than the change in the electrode spacing. The final capacitance of the sensor can be increased from pF to nF, which can effectively improve the sensitivity of the sensor. The addition of a COS chain extender can make ILs better dispersed in the polymer network, thus forming a larger EDL capacitance and effectively improving the sensitivity of the sensor. As shown in Figure 6f, the sensor has an excellent sensitivity and detection range compared with other capacitive sensors based on the photosensitive resin [32,37,38,39,47,48].

Next, other properties of the sensor are tested. As shown in Figure 6a, a pressure of 3 kPa was applied to the sensor by the Instron universal testing machine. The sensor had a fast response time of 51 ms and returned to the initial value within 85 ms. As shown in Figure 6b, it has a reliable response to gradually increase the force of 20 kPa on the sensor. Repeated stepped small stress and large stress (1, 10, 20, 50, and 100 kPa) applied to the sensor also have a reliable pressure response (Figure 6c). In order to further evaluate the mechanical durability of the sensor and meet its long-term application, the sensor was tested for 1000 loading/unloading cycles at about 1 kPa. As shown in Figure 6d, the signal has basically no drift and fluctuation, and the signal value is only attenuated by 7.13% under 1000 compression cycles. As shown in Figure 6e,f, the sensor can detect the beat of the human pulse and different physiological signals of the human throat. The sensor is packaged with medical tape and attached to the pulse and throat of the human body, respectively. The enlarged figure in Figure 6e shows that the sensor can clearly identify the percussion (P) wave, the tidal (T) wave, and the dicrotic (D) wave of the pulse. Figure 6f shows that the sensor can distinguish three different models of deep breathing, swallowing, and coughing. As shown in Figure S13, by continuously measuring the cough pattern multiple times, the sensor can clearly detect the changes in the throat. This is of great significance to human health monitoring.

## 4. Conclusions

In summary, an ion composite photosensitive resin for DLP 3D printing was prepared in this work. The chitooligosaccharide chain extender was introduced to achieve the compatibility between the photosensitive resin and the ILs. The Young’s modulus of the cured ion composite photosensitive resin can reach 0.85 MPa, and the horizontal printing resolution can reach 50 μm. This photosensitive resin can print various complex three-dimensional structures. By adjusting the structure and density ratio of the dielectric layer, the sensitivity of the sensor can reach 32.62 kPa^−1^. At the same time, the sensor exhibits a response time of 51 ms and good mechanical durability, and it can generate reliable electrical signals under 1000 cycles. The sensor can also monitor the weak pressure signals of the pulse and throat. More importantly, the ion composite photosensitive resin can be used for DLP 3D printing, providing more possibilities for structural design for the preparation of wearable sensors.

## Data Availability

The data are contained within the article and Supplementary Materials.

## Supplementary Materials

The following supporting information can be downloaded at: https://www.mdpi.com/article/10.3390/s25051348/s1, Figure S1: (a) FTIR spectra of two different materials after UV curing; (b) FTIR spectra of PUA+COS after UV curing and heat curing; Figure S2: The reaction of PUA and COS under heat curing; Figure S3: The contact angles between three different ILs and two different photosensitive resins; Figure S4: (a) SEM image and (b) EDX image of the surface of TYPE-C structure 3D-printed by ion composite photosensitive resin; Figure S5: FTIR spectra of photosensitive resin without ILs and ion composite photosensitive resin; Figure S6: (a) Mechanical performance test system; (b) The type 3 sample parameters specified in GB/T 1040.3-2006 (unit: mm); Figure S7: The conductivity of the material changes with the content of [EMIM][TFSI]; Figure S8: Mechanical properties of block samples before and after fatigue test; Figure S9: The change of weight of ion composite photosensitive resin with temperature; Figure S10: The change in E_c_ and D_p_ with the content of [EMIM][TFSI]; Figure S11: The variation of stress with strain in different structures under compression; Figure S12: The sensitivity of sensors with TYPE-C dielectric layers of different density ratios; Figure S13: Real-time monitoring of cough in the human throat.
