# Supplementary material for: Preparation of Ion Composite Photosensitive Resin and Its Application in 3D-Printing Highly Sensitive Pressure Sensor
Source: Sensors (Basel). 2025 Feb 22;25(5):1348. doi: 10.3390/s25051348 (PMC11902503; doi:10.3390/s25051348)
Supplement: Supplementary file 1 [file sensors-25-01348-s001.zip › sensors-3471817-supplementary.pdf]

Supporting Information

Preparation of Ion Composite Photosensitive Resin and Its Application in 3D-Printing Highly Sensitive Pressure Sensor

Tong Guan [1], Huayang Li [2], Jinyun Liu [3,4,5], Wuxu Zhang [3,4,5], Siying Wang [3,4,5], Wentao Ye [3,4,5], Baoru Bian [3,4], Xiaohui Yi [3,4], Yuanzhao Wu [3,4], Yiwei Liu [3,4], Juan Du [1,*], Jie Shang [3,4,*] and Run-Wei Li [3,4,*]

[1] School of Materials Science and Engineering, Shanghai University, Shanghai 200072, China; guantong@nimte.ac.cn
[2] Yongjiang Laboratory, Ningbo 315201, China; huayang-li@ylab.ac.cn
[3] CAS Key Laboratory of Magnetic Materials and Devices, Ningbo Institute of Materials Technology and Engineering, Chinese Academy of Sciences, Ningbo 315201, China; liujinyun@nimte.ac.cn (J.L.); zhangwuxu@nimte.ac.cn (W.Z.); wangsiying@nimte.ac.cn (S.W.); yewentao@nimte.ac.cn (W.Y.); bianbr@nimte.ac.cn (B.B.); yixiaohui@nimte.ac.cn (X.Y.); wuyz@nimte.ac.cn (Y.W.); liuyw@nimte.ac.cn (Y.L.)
[4] Zhejiang Province Key Laboratory of Magnetic Materials and Application Technology, Ningbo Institute of Materials Technology and Engineering, Chinese Academy of Sciences, Ningbo 315201, China
[5] College of Materials Science and Opto-Electronic Technology, University of Chinese Academy of Sciences, Beijing 100049, China
* Correspondence: jdu-case@hotmail.com (J.D.); shangjie@nimte.ac.cn (J.S.); runweili@nimte.ac.cn (R.-W.L.)

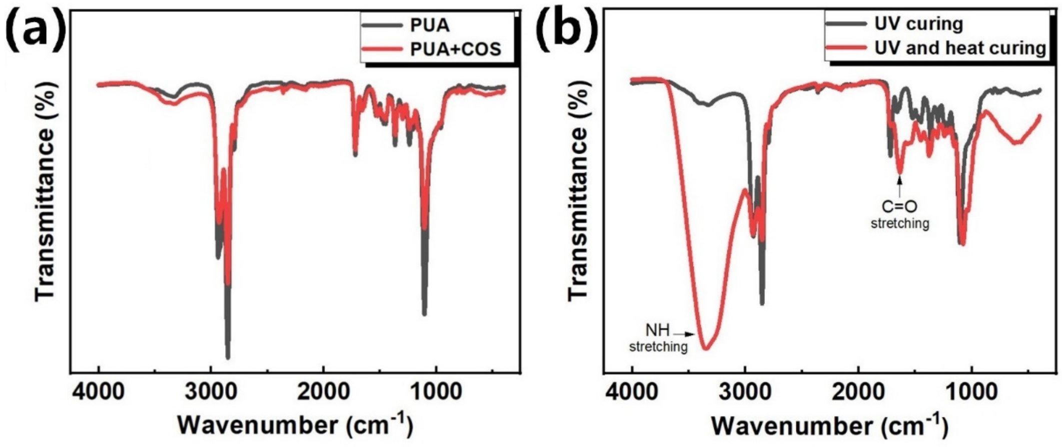

Figure S1. (**a**) FTIR spectra of two different materials after UV curing; (**b**) FTIR spectra of PUA+COS after UV curing and heat curing.

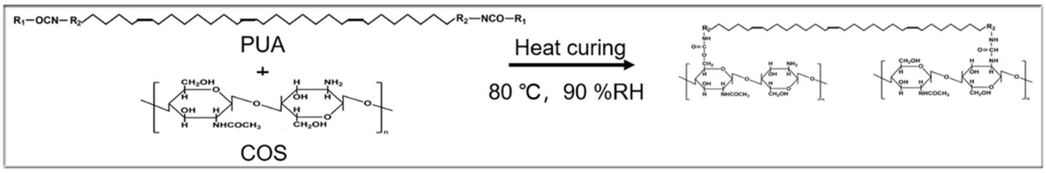

Figure S2. The reaction of PUA and COS under heat curing.

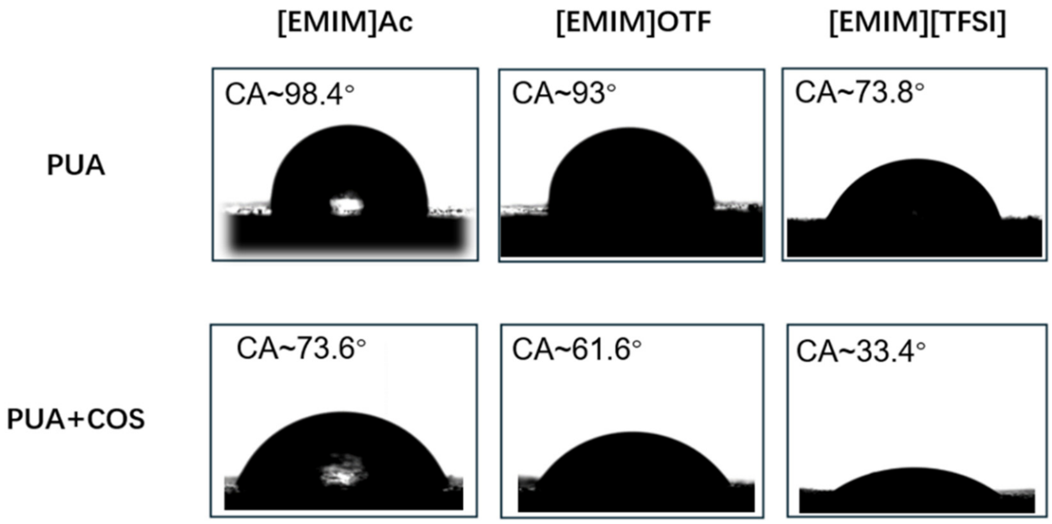

Figure S3. The contact angles between three different ILs and two different photosensitive resins.

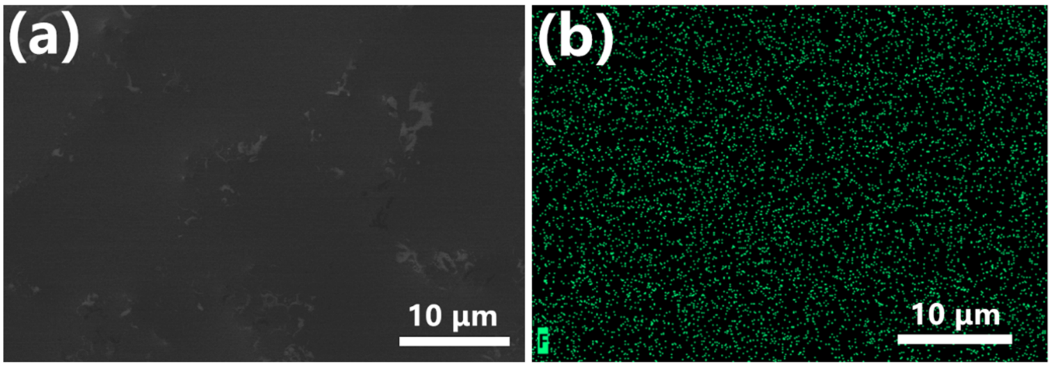

Figure S4. (**a**) SEM image and (**b**) EDX image of the surface of TYPE-C structure 3D-printed by ion composite photosensitive resin.

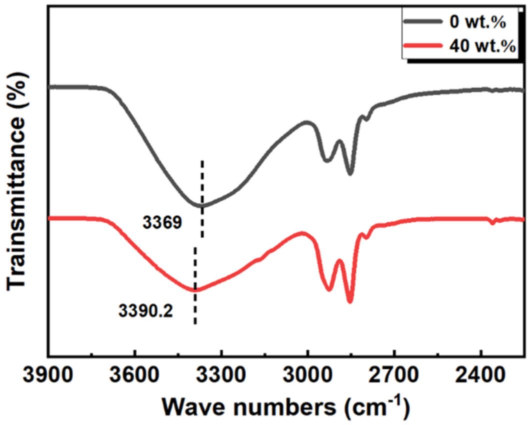

Figure S5. FTIR spectra of photosensitive resin without ILs and ion composite photosensitive resin.

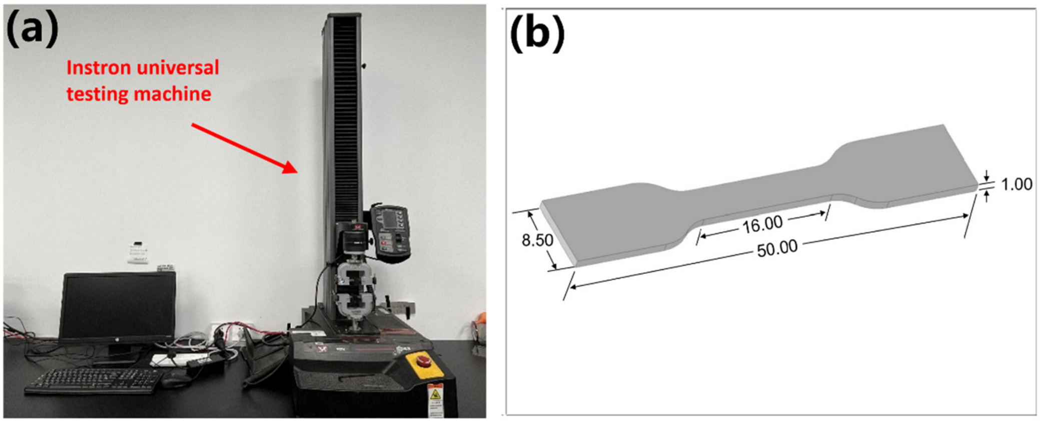

Figure S6. (**a**) Mechanical performance test system; (**b**) The type 3 sample parameters specified in GB/T 1040.3-2006 (unit: mm).

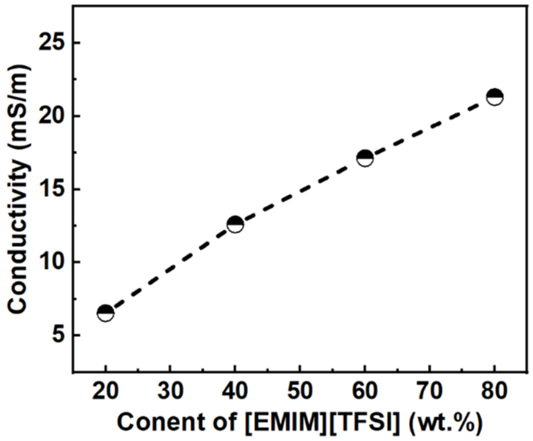

Figure S7. The conductivity of the material changes with the content of [EMIM][TFSI].

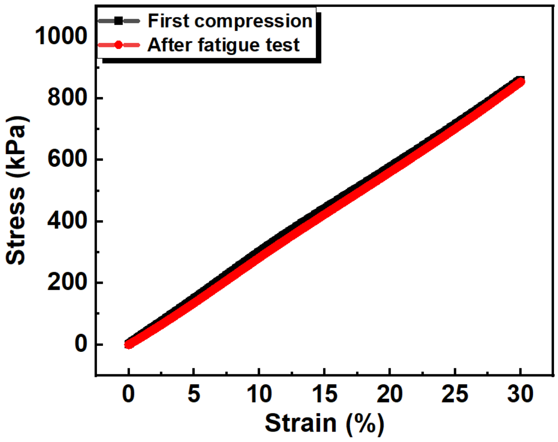

Figure S8. Mechanical properties of block samples before and after fatigue test.

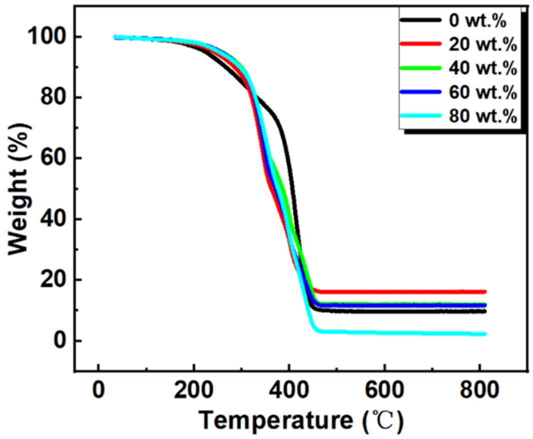

Figure S9. The change of weight of ion composite photosensitive resin with different content of ILs with temperature.

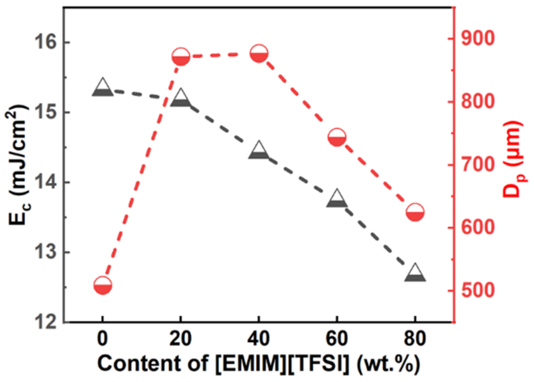

Figure S10. The change of E_c and D_p with the content of [EMIM][TFSI].

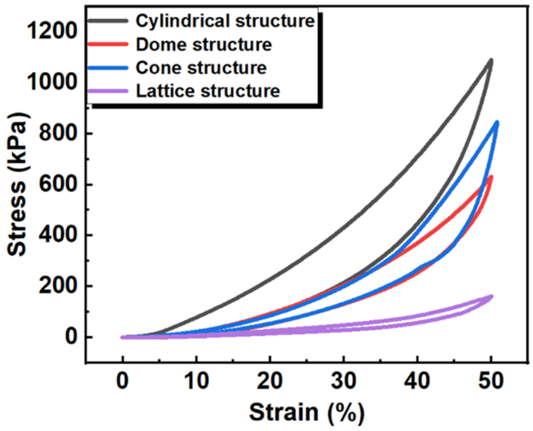

Figure S11. The variation of stress with strain in different structures under compression.

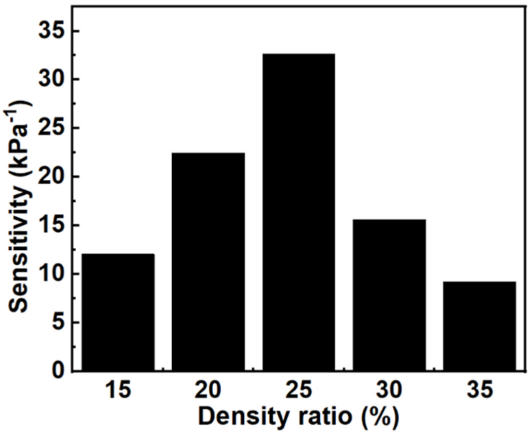

Figure S12. The sensitivity of sensors with TYPE-C dielectric layers of different density ratios.

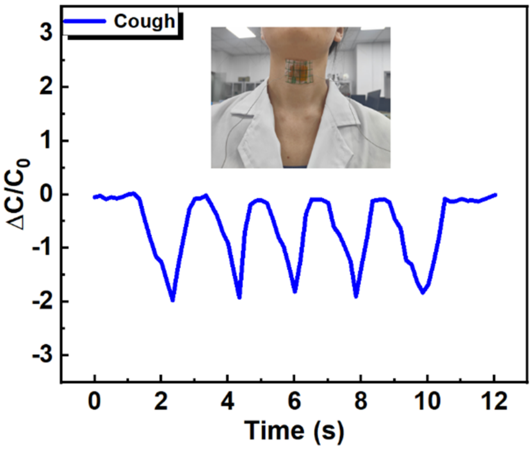

Figure S13. Real-time monitoring of cough in the human throat.
